# Supplementary material for: NIR-II fluorescence projection technology for augmented-reality surgical navigation
Source: Natl Sci Rev. 2026 Mar 13;13(9):nwag158. doi: 10.1093/nsr/nwag158 (PMC13166876; doi:10.1093/nsr/nwag158)
Supplement: nwag158_Supplemental_Files [file nwag158_supplemental_files.zip › nwag158_revised_SI-0312.pdf]

Supplementary Materials for

• NIR-II Fluorescence Projection Technology for Augmented Reality Surgical Navigation

Yuhuang Zhang *et al.*

*Corresponding author. Email: qianjun@zju.edu.cn

This PDF file includes:

Supplementary materials and methods
Fig. S1 to S8
Table S1 to S2

1. Supplementary materials and methods

Testing of System’s Resolution Limit

USAF 1951 resolution charts were used as standard test samples. These charts, composed of chrome-coated glass with light-transmitting patterns etched into the chrome layer, contain groups of horizontal and vertical lines with varying sizes. The spatial resolution of the system was evaluated based on the finest set of lines that could be clearly distinguished. The resolution charts were placed directly on the surface of the NIR light source. At a working distance of 28 cm, the NIR-II fluorescence projection system was employed to perform imaging and projection, after which the projected images were frozen. The resolution charts were then replaced with white paper to serve as the projection screen. A high-resolution, visible-light-sensitive camera (Bigeye, Hikvision, China) was used to capture the projected patterns. The clarity of the captured projection images served as an indicator of the system's resolution, which was influenced collectively by factors such as equipment alignment, the performance of the InGaAs camera, the LCD panel resolution, and the optical resolution of the objective lens.

Characterization of System’s Positioning Error

Checkerboard patterns with edge lengths of 9.57 mm were printed and used for system positioning accuracy testing. First, their bright-field images were captured using a fixed visible-light-sensitive camera. The printed checkerboards were then illuminated with halogen lamps containing NIR-II spectral components, allowing the NIR-II fluorescence projection system to image the patterns and project corresponding green pseudocolored checkerboards back onto the surface. After freezing the projected images, the printed checkerboards were replaced with white paper to serve as a projection screen. The visible-light-sensitive camera then captured the projected checkerboard patterns on the paper. The positioning accuracy of the system was evaluated by comparing the corner points of the original printed checkerboards and those of the projected patterns. The visible-light-sensitive cameras used had RGBW pixel arrangements, and the W (white) channels were extracted for analysis. Corner point detection was performed using MATLAB, and the pixel-wise distances between corresponding corner point pairs in the original and projected images were calculated. Knowing the physical edge length of the checkerboard (9.57 mm), the pixel distances were converted into millimeters. Given that surgical targets often have irregular, undulating surfaces, the NIR-II fluorescence projection system is required to maintain high positioning accuracy within its depth of field. Therefore, after fixing the system position, the checkerboards were repositioned at various depths within the depth of field to evaluate positioning errors under different spatial conditions.

Materials Preparation

Indocyanine Green (ICG) for animal experiments was obtained from Meilunbio (Dalian, China), while clinical-grade ICG was purchased from Dandong Yichuang Pharmaceutical Co., Ltd. and dissolved in the provided sterile water for injection. Polymer dots (Pdots), PbS/CdS quantum dots (QDs), and aggregation-induced emission dots (AIE dots) were synthesized according to previously published protocols [11-13]. Deionized water was used as the solvent in all experiments, except those involving clinical imaging.

Animal Handling

All animals were obtained from the Zhejiang University School of Medicine. The experimental subjects included 20 g female ICR mice, 300 g female Sprague-Dawley (SD) rats, and 2 kg New Zealand white rabbits. To evaluate the projection visibility on various biological tissues, the NIR-II fluorescence projection system was configured to project seven-pointed stars at maximum brightness. Under indoor lighting conditions of approximately 2000 lux, the projection patterns were directed onto various colored rat tissues, including the abdominal skin, fascia, kidney, liver, intestine, and blood. The resulting images were captured using mobile phones. For imaging the leg lymphatic system and retroperitoneal lymph nodes of mice, 100 µL of ICG aqueous solution (1 mg/mL) was subcutaneously injected into the hind paw pads. The ICG subsequently traveled through the lymphatic vessels, sequentially staining the popliteal and retroperitoneal lymph nodes. Similarly, to visualize the retroperitoneal and axillary lymph nodes in rats, 500 µL of ICG solution (1 mg/mL) was injected into the hind and forepaw pads, respectively. For vascular imaging in rabbits, 2 mL of ICG aqueous solution (1 mg/mL) was intravenously injected into the ear vein after the animal was anesthetized. To image and project the rabbit's leg lymphatic system, an equal volume of ICG solution was injected into the paw pads. Prior to all experiments, the imaging sites of all animals were depilated using depilatory cream to reduce interference from fur.

Sterilization and Intraoperative Setup

To eliminate potential optical interference and signal attenuation associated with transparent sterile drapes, we adopted a "split sterilization" strategy for the clinical trials. The main body of the NIR-II fluorescence projection system was shielded using a standard disposable sterile drape. In contrast, the custom dual-channel objective lens was left exposed but underwent preoperative terminal sterilization using Hydrogen Peroxide Low-Temperature Plasma (VH_2O_2). This specific sterilization modality was selected for its low-temperature and dry characteristics, which effectively ensured medical-grade sterility while preserving the integrity of the precision optical coatings and leaving no toxic residue.

Patient Recruitment and Clinical Data Collection

Volunteers were recruited from patients undergoing treatment for diabetic foot and lymphedema at the affiliated hospital of Zhejiang University. After being informed and fully understanding the study procedures, the volunteers participated in clinical trials utilizing our NIR-II fluorescence projection system. Patients with diabetic foot received intravenous injections of ICG (3 mL, 2.5 mg/mL). In addition, a healthy volunteer was recruited and received a single-point subcutaneous injection of ICG at the wrist, followed by NIR-II imaging and projection for lymphatic visualization of the arm and wrist. The detailed clinical baseline characteristics of the recruited subjects are summarized in Table S2.

2. Figures

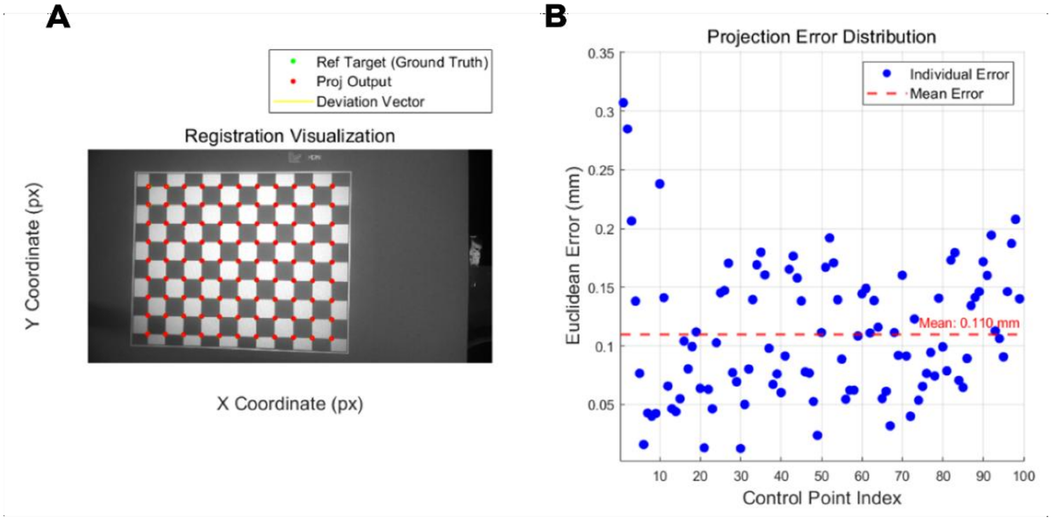

Fig S1. (A) Registration visualization. The projected fiducials (red dots, Proj Output) are overlaid on the ground-truth physical checkerboard corners (green dots, Ref Target). The tight spatial overlap renders both the corners (green dots) and the deviation vectors (yellow lines) virtually imperceptible, visually confirming the negligible projection error and high alignment fidelity. (B) Error distribution analysis. The scatter plot displays the Euclidean error for each validated control point across the field of view. The system achieves a mean projection error of 0.110 mm (red dashed line), confirming robust sub-millimeter precision for surgical navigation.

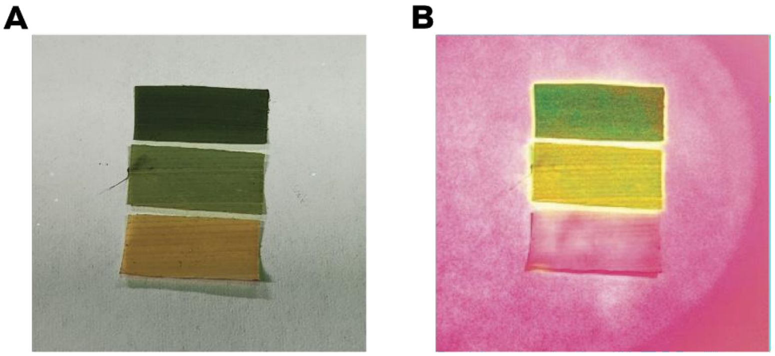

Fig S2. Demonstration of chlorophyll NIR-II fluorescence detection in maize leaves using the projection system. (A) Photographs of maize leaves representing distinct developmental stages: emerging (top), mature (middle), and senescent (bottom). (B) Fluorescence imaging and projection under ~690 nm excitation using the developed system. The tender green and mature leaves exhibit clear NIR-II fluorescence, visualized here as projected green light. In contrast, the yellowed leaf, exhibiting significantly reduced chlorophyll content, shows negligible fluorescence and appears like the background.

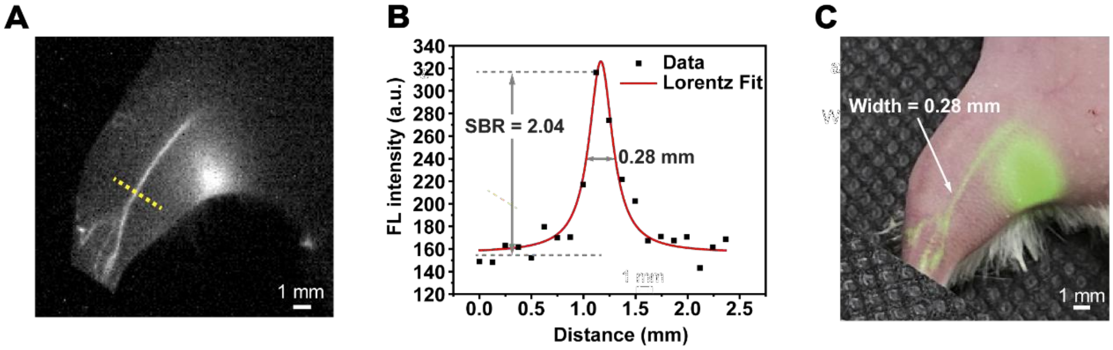

Fig S3. In vivo mapping of vascular system in mouse using the projection system. (A) A NIR- II fluorescence image of ICG-labeled lymph node and blood vessels. (B) Intensity profile along yellow dashed line in (A). Lorentzian fitting reveals a blood vessel width (FWHM) of 0.28 mm with SBR = 2.04. (C) The projected pseudocolored fluorescence image demonstrating precise co-localization of lymph node and submillimeter blood vessels (0.28 mm width).

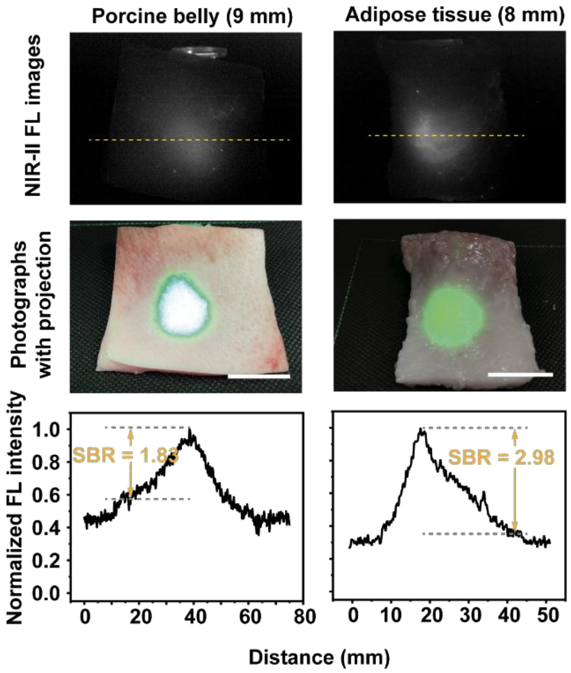

Fig S4. Porcine belly (9 mm) and adipose tissue (8 mm) were overlaid on microcentrifuge tubes containing 0.1 mg/mL ICG solution. The NIR-II fluorescence projection system maintained fluorescence localization capability at these depths with sustained SBRs of 1.83 and 2.98 respectively, as well as precise projection.

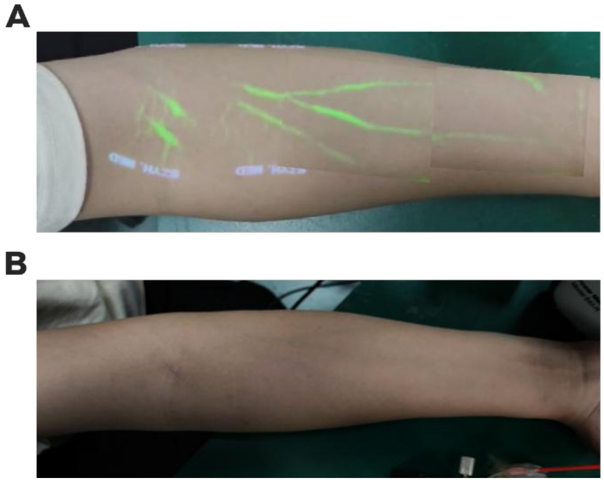

Fig S5. Comparison of vascular and lymphatic visualization in the human forearm. (A) Projection of superficial venous structures on the right forearm of a healthy volunteer using a commercial vein imaging system, revealing a structure distinct from the lymphatic vessels shown in Fig 6I. (B) Bright-field image of the same forearm. Note: The limited field of view (FOV) of the commercial imaging system necessitated image stitching to generate the complete projection.

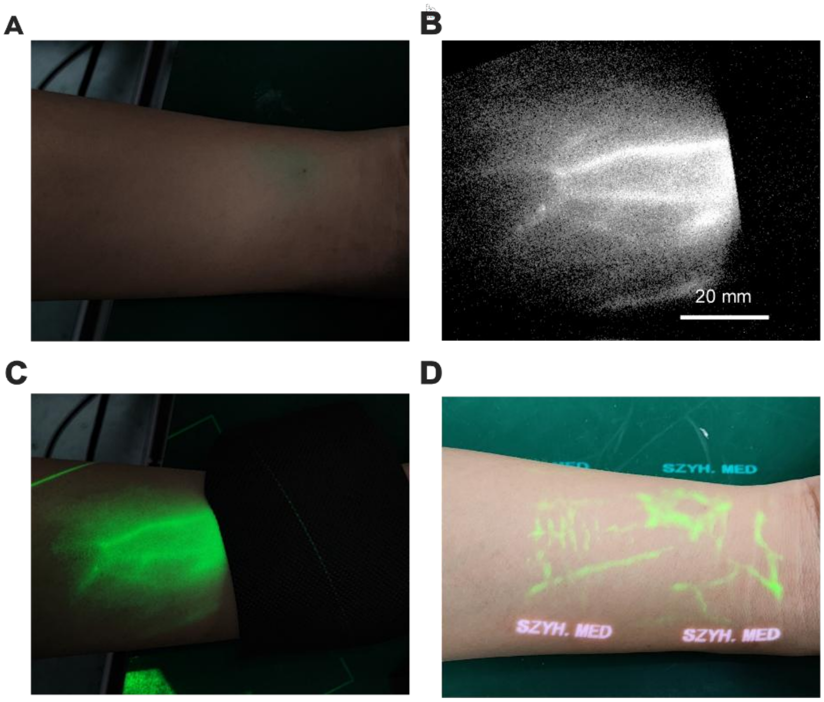

Fig S6. Lymphatic mapping following subcutaneous injection of ICG. (A) Bright-field image of the injection site at the wrist. (B) NIR-II fluorescence image showing the lymphatic vasculature (injection site masked to prevent signal oversaturation). (C) Projected pseudocolored NIR-II fluorescence image illustrating lymphatic channels. (D) Comparative projection using a commercial vein imaging system (VeinViewer® Flex), confirming distinct spatial patterns

between the vascular (D) and lymphatic (C) systems. Imaging was performed 4 hours after ICG injection (0.5 mL, 0.1 mg/mL).

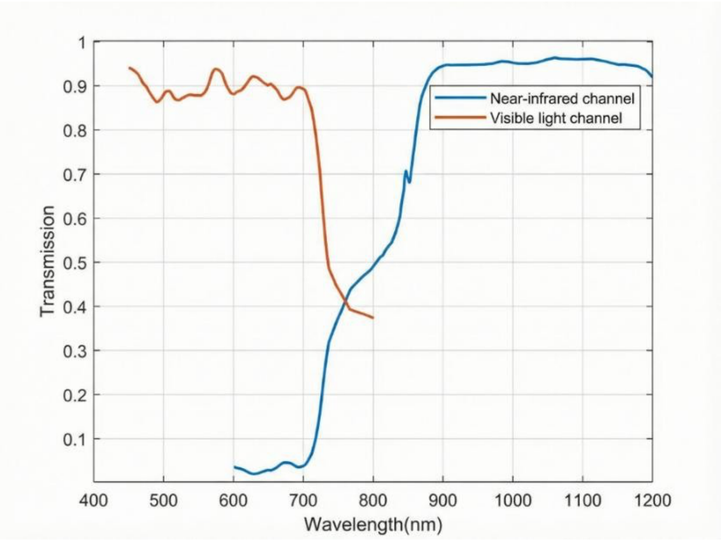

Fig S7. Spectral transmittance characterization of the customized co-axial dual-channel objective. By optimizing the lens coatings, the objective achieves high transmittance (>85%) in the visible projection band (400–700 nm). Crucially, the design maintains superior transmittance (>95%) in the 900–1200 nm range, effectively covering the emission spectrum of ICG and many NIR-II fluorophores.

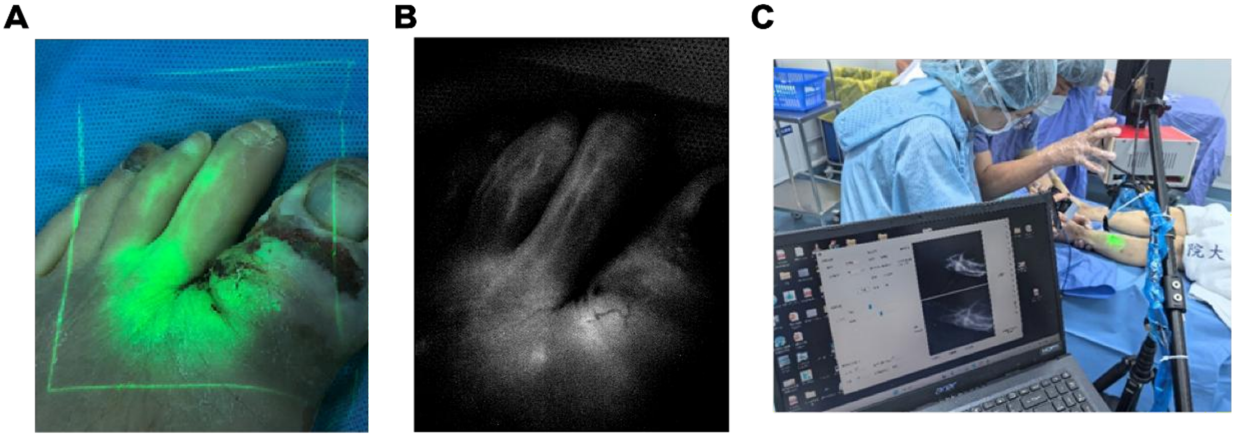

Fig S8. Clinical trial in another diabetic foot patient. (A) Bright-field image of the dorsal foot in Patient 3 (diabetic foot with early-stage superficial ulcer) following intravenous injection of ICG, overlaid with the projected green pseudocolored NIR-II fluorescence image. (B) Corresponding raw NIR-II fluorescence image. A clear distinction in vascular morphology is observed: while healthy toes display a sharp microvascular network, the inflamed hallux exhibits diffuse fluorescence with blurred boundaries. (C) Deployment of the NIR-II fluorescence projection system in a standard operating room.

Key Parameters	Specifications
Imaging Sensor	InGaAs camera (SD640, 640×512 pixels, 15 μm pixel pitch)
Spatial Resolution	~220 μm
Field of View (FOV)	10 cm × 13 cm (at 28 cm WD)
Working Distance (WD)	24–100 cm
Positioning Error	< 0.15 mm (at 28 cm WD)
Detection Capability	0.1 mg/mL ICG detectable at 9 mm depth

Table S1. Key technical specifications of the NIR-II fluorescence projection system.

Characteristic	Patient 1 (Figure 6)	Patient 2 (Figure 6)	Patient 3 (Supplementary Figure 8)
Age (years)	65	57	48
Gender	Male	Male	Male
Weight (kg)	65.6	60	54
BMI (kg/m^2)	24.1	20.8	15.6
Smoking History	Yes	Yes	Yes
Alcohol Consumption	No	Yes	No
Blood Pressure (mmHg)	129/78	145/101	127/78
Blood Glucose (mmol/L)	9.7	10.2	17.8
Diagnosis	**Diabetic foot with deep ulcer**	**Diabetic foot with gangrene**	**Diabetic foot with superficial ulcer**
Wagner Classification	Stage 2	Stage 3	Stage 1
Disease Duration (years)	3	5	1
ICG dosage (mL, 2.5 mg/mL)	3	3	3
Operation time (min)	45	120	30

Table S2. Characteristics and preoperative diagnosis of included patients.
